# High Rates of Organ Preservation in Rectal Cancer with Papillon Contact X-ray Radiotherapy: Results from a Swiss Cohort

**DOI:** 10.3390/cancers16132318

**Published:** 2024-06-25

**Authors:** Cristina Picardi, Francesca Caparrotti, Michael Montemurro, Daniel Christen, Nora-Brunner Schaub, Marie Fargier-Voiron, Laetitia Lestrade, Jeremy Meyer, Guillaume Meurette, Emilie Liot, Daniel Helbling, Jan Schmidt, Jean-Pierre Gutzwiller, Marco Bernardi, Oscar Matzinger, Frederic Ris

**Affiliations:** 1 Klinik Bethanien, Swiss Medical Network, 8044 Zürich, Switzerland; cpicardi@genolier.net (C.P.);; 2 Hirslanden Klinik, Witellikerstrasse 40, 8032 Zürich, Switzerland; 3 Clinique Générale-Beaulieu, Swiss Medical Network, 1206 Geneva, Switzerland; fcaparrotti@beaulieu.ch; 4 Clinique de Genolier, Swiss Medical Network, 1272 Genolier, Switzerlandomatzinger@genolier.net (O.M.); 5 Geneva University Hospital and Medical School, 1205 Geneva, Switzerland; 6 Medical Oncology, GITZ—Gastrointestinales Tumorzentrum Zürich, 8038 Zürich, Switzerland; 7 Visceral Surgery, GITZ—Gastrointestinales Tumorzentrum Zürich, 8038 Zürich, Switzerland; 8 Gastroenterology, GITZ—Gastrointestinales Tumorzentrum Zürich, 8038 Zürich, Switzerland

**Keywords:** rectal cancer, organ preservation, radiotherapy dose escalation, contact X-ray radiotherapy

## Abstract

**Simple Summary:**

Our study in Switzerland since 2015 explored the Papillon device for treating rectal cancer, aiming to enhance organ preservation in an upfront strategy. We integrated Papillon’s low-energy X-ray boost with standard radiotherapy (RT), observing 96% organ preservation and an 8% local relapse rate over a median 43-month follow-up. Crucially, no patients experienced severe (grade 3 or higher) toxicities. This approach effectively escalates RT dose with minimal impact on surrounding tissues, offering a viable alternative to surgery. Our findings underscore the device’s efficacy in achieving high local remission rates and supporting long-term organ preservation, contributing valuable insights to advance rectal cancer treatment strategies.

**Abstract:**

Rectal cancer typically necessitates a combination of radiotherapy (RT), chemotherapy, and surgery. The associated functional disorders and reduction in quality of life have led to an increasing interest in organ preservation strategies. Response strongly correlates with RT dose, but dose escalation with external beam remains limited even with modern external beam RT techniques because of toxicity of the surrounding tissues. This study reports on the use of Papillon, an endocavitary Radiotherapy device, in the treatment of rectal cancer. The device delivers low energy X-rays, allowing for safe dose escalation and better complete response rate. Between January 2015 and February 2024, 24 rectal cancer patients were treated with the addition of a boost delivered by Papillon to standard RT, with or without chemotherapy, in an upfront organ preservation strategy. After a median follow-up (FU) of 43 months, the organ preservation rate was 96% (23/24), and the local relapse rate was 8% (2/24). None of our patients developed grade 3 or more toxicities. Our results demonstrate that the addition of Papillon contact RT provides a high rate of local remission with sustained long-term organ preservation, offering a promising alternative to traditional surgical approaches in patients with rectal cancer.

## 1. Introduction

Colorectal cancer is the third most common cancer in men and the second most common in women in Switzerland and worldwide [1,2]. In Switzerland, around 4500 new cases of colorectal cancer are diagnosed every year [1], of which 20–30% occur in the rectum [2,3,4]. Although the incidence in younger patients (<50 years) has increased in recent years, the incidence of rectal cancer increases proportionally with age and peaks at an age of 70 years [1,5,6,7].

Except in the rare cases where the tumor is diagnosed at a very early stage and can be operated on directly, most rectal cancers require a combination of radiotherapy (RT), chemotherapy, and surgery. Radical proctectomy based on the concept of total mesorectal excision (TME) is considered the standard of care for cT2-3 cN0-3 cM0 tumors, usually performed 6–12 weeks or more after the completion of preoperative chemoradiotherapy (CRT) [8]. However, more than half of the patients after rectal resection suffer from a functional disorder, such as incontinence, urgency, and frequent bowel movements, with a general reduction in quality of life [9]. The advanced age at diagnosis complicates multimodal patient management with regard to comorbidities and cumulative risk factors. Mortality in elderly patients can be up to 30% [10].

A watch-and-wait strategy was introduced in the late 1990s in patients obtaining a complete clinical response after CRT [7]. Since then, organ preservation in rectal cancer has been a topic of growing interest worldwide. Nevertheless, despite very encouraging data, one-fourth of patients experience relapse [11].

**Figure 1:**
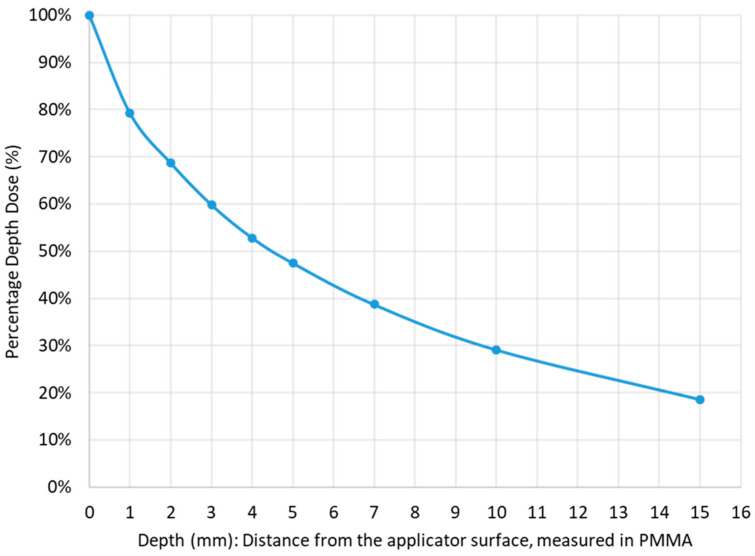
Dose fall-off curve. At 4.4 mm, the dose is divided by 2 compared to the surface dose.

**Figure 2:**
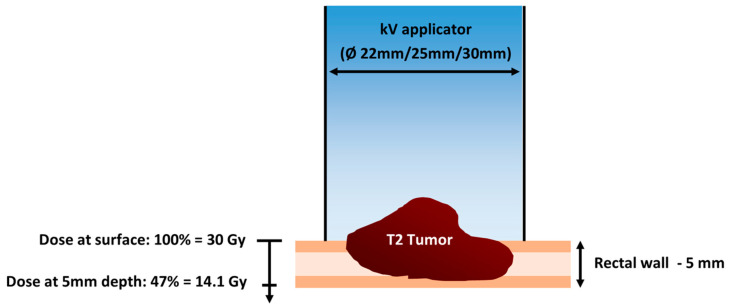
Schematic representation of Papillon treatment and dose distribution in the depth.

Studies have shown that the RT dose correlates significantly with the pCR rate [12]. In fact, doses higher than 80–90 Gy would be required to achieve complete sterilization of the tumor [13]. This is not possible even with modern RT techniques, as serious early and late toxicities could occur in the surrounding healthy organs [14]. However, this dose escalation is safely achieved with the Papillon^TM^ (Ariane Medical Systems, Alfreton, UK), an endocavitary contact RT device delivering low-energy X-rays (50 kV, HVL 0.53 mm, 2.7 mA), which have a rapid dose fall-off, offering a favorable toxicity profile (Figure 1). With each application, the tumor is treated with a very high dose at its surface and is destroyed layer by layer in a very small volume of tissue, typically <5 cm^3^ (Figure 2). Papillon therapy is not only ablative but is also an adaptive RT technique as the dose and the diameter of the applicator are adjusted to the thickness and size of the tumor during each treatment.

Despite its proven efficacy, Papillon contact RT remains quite unknown, with only about 12 Papillon devices currently installed in the UK, France, Denmark, Sweden, the Netherlands, and Switzerland. 

We report our national results of rectal cancer patients treated with the Papillon device. Informed consent was obtained from all subjects involved in this study.

## 2. Materials and Methods

Between January 2015 and February 2024, we treated 88 rectal cancer patients with Papillon contact RT in different settings. However, in this report, we decided to exclude patients that were treated in a palliative setting (n = 7), before a planned surgery (n = 4), as salvage treatment (n = 4), and as an adjuvant treatment after a resection of a T1 cancer that revealed poor prognostic features (n = 6), along with those lost to follow up (n = 20) and those with an insufficient (<12 months) follow up (n = 23).

We assessed the organ-preservation rate and the local control of 24 patients treated with an upfront organ preservation strategy and a minimum FU of 12 months. 

Patient characteristics were retrospectively collected from a prospective database. 

All patients were discussed and managed by a multidisciplinary panel of experts. All patients were deemed operable. All patients provided written consent to proceed with an organ preservation strategy. 

Staging at diagnosis consisted of a local examination that included a digital rectal examination, a rigid rectoscopy, multiparametric pelvic MRI, and an endosonography if deemed necessary. A complete colonoscopy was required to exclude a second synchronous tumor. To complete the local staging, a CT scan of the thorax and abdomen (or a PET CT in some cases) was performed to exclude distant metastasis. The CEA tumor marker was measured at baseline.

The first assessment was performed at 6 weeks after the end of the Papillon RT to judge the clinical response using DRE and rectoscopy. At 3 months, patients underwent DRE, rectoscopy (+/− endosonography), MRI, CEA measurement, and biopsy in dubious cases. Surgery was indicated in cases of non-response at 3 months.

In cases of complete response at 3 months, patients underwent the abovementioned exams at a 3-month interval for the first 2 years and every 6 months thereafter. A chest and abdominal CT-scan was required every 6 months for the first 2 years, then annually and a colonoscopy once per year.

Toxicity was assessed at every FU visit using CTCAE version 4.

Technically, the application of the Papillon is similar to a rigid rectoscopy; an enema was given 30–40 min before each treatment. Even if general anesthesia was not required, we offered sedation for patient comfort. The treatment was always performed on an outpatient basis. Papillon boost was delivered with an interval of 1–2 weeks. The common prescription dose for a >T2 tumor is 3 fractions of 30 Gy at the surface. For 12 patients, the dose per fraction was adapted to the local situation at the moment of treatment, with lower doses being prescribed for near-complete response or macroscopically sane mucosa. We standardized the dose prescription to 3 × 30 Gy according to the OPERA trial protocol, after joining the study in September 2018. The applicator diameter (2.2 cm, 2.5 cm, or 3 cm) was chosen based on the size of the tumor at the time of treatment. As common practice, for patients presenting with small tumors (<than 3 cm), Papillon therapy was performed before RT (n = 7). The remaining patients with larger tumors had their Papillon therapy after a standard CRT and a median interval of 3 weeks (2–4). 

All patients received external beam radiotherapy using imaging planning; as a standard of care, the target volume included the visible tumor, the mesorectum, the presacral, and the internal iliac nodal structures. The S2 or S3 interspace was the clinical target volume’s upper limit. Radiotherapy was delivered either as 3D conformal radiotherapy or intensity modulated radiotherapy with a photon beam of 6 mV or more and image guidance at a dose of 45 Gy in 25 fractions over 5 weeks. Concurrent chemotherapy was given to all patients and consisted of oral capecitabine (825 mg/m^2^ twice a day) over 5 weeks from the first day that patients received their radiotherapy.

### Statistical Analysis

We performed a Kaplan–Meier test for local relapse and TME-free survival. Local relapse was defined as any visible tumor observed during rectoscopy and/or MRI and confirmed by histology after achieving a complete response. TME-free survival was defined as organ preservation at 12 months.

## 3. Results

All of our patients presented a complete clinical response at the first assessment at 6 weeks after the treatment had been completed. Most patients presented with low rectal cancer tumors (Ra) (n = 16), 4 patients with ultra-low (Rb) located lesions, defined as being <5 cm from the anal verge, and 4 patients had middle rectal cancer (Rs). Patients’ characteristics and treatment are summarized in Table 1. Four patients had T1 tumors, 9 patients had T2 tumors, 10 patients had T3 tumors, and 1 patient had a T4 tumor. Most patients were node-negative, with a minority having cN1 (n = 4), and only one patient presented with cN2.

**Figure 3:**
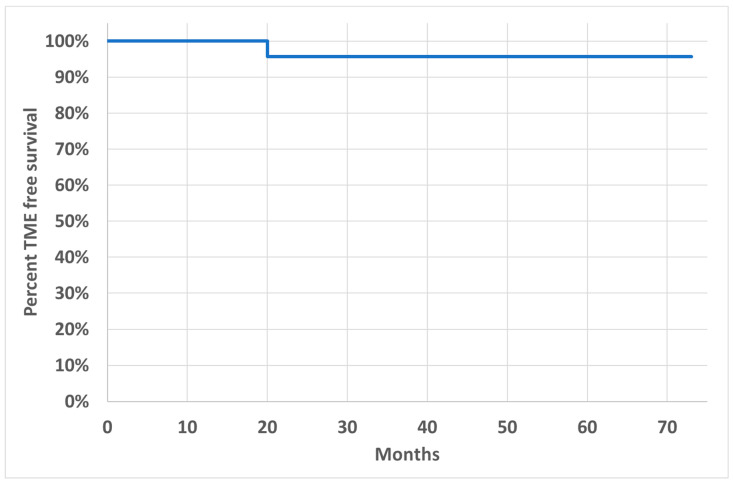
Kaplan–Meier analysis for TME-free survival. Median follow-up: 43 months.

**Figure 4:**
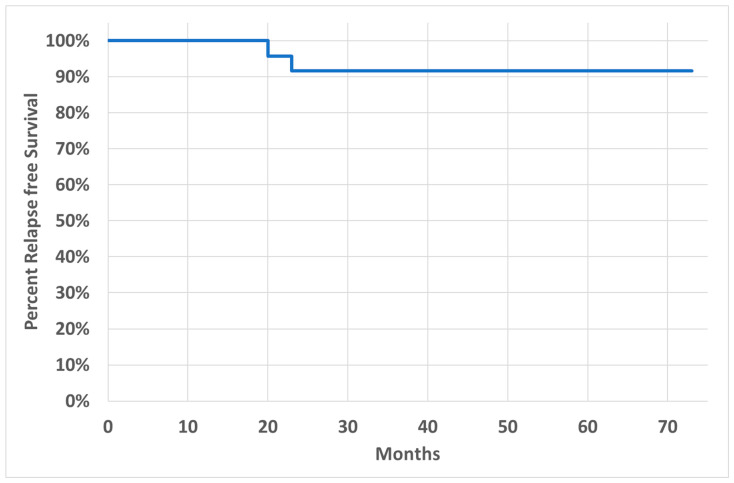
Kaplan–Meier analysis for relapse-free survival. Median follow-up: 43 months.

After a median FU of 43 months (range 13–73), the organ preservation rate was 96% (23/24) (Figure 3). The local relapse rate was 8% (2/24) (Figure 4). 

Two patients had a local relapse detected during follow-up. Of these, one presented with initial stage T3 N1 M0 and relapsed 18 months after the end of the treatment and consequently underwent radical surgery (TME), remaining disease-free thereafter. 

The second patient presented a small local regrowth at 11 months after the last treatment and was salvaged with a local excision, allowing the preservation of the rectum. Surprisingly, this patient had a very favorable initial stage (T2 N0 M0) and presented an excellent clinical response already after only two Papillon fractions. It could be speculated that since no macroscopic tumor could be targeted at the last fraction, a possible target miss would explain this relapse.

During the follow up period, one patient died of severe pulmonary disease. The survival rate was 96% (23/24). There was no correlation with the comorbidities of the patients.

No patients developed distant or pelvic metastasis. Ten patients achieved long-term (>3 year) organ preservation.

Papillon boost toxicities were recorded independently of the side effects of EBRT (external beam radiotherapy) and/or chemotherapy and were considered acute when symptoms occurred within the Papillon RT treatment period and/or in the three months after the end of the last fraction. Treatment toxicities are summarized in Table 2 and Table 3. Ten patients had grade 1 toxicity. Impaired bowel movements, meteorism, and mucous discharge were the most frequently reported symptoms, followed by minor rectal bleeding. Only four patients had grade 2 toxicity, reporting perianal pain that needed medication with topical steroids. Late side effects (beyond 3 months after the end of treatment) were rare. One patient presented grade 1 rectal bleeding 7 months after the end of Papillon RT which spontaneously resolved itself, and four patients presented rectal bleeding that needed cauterization (grade 2) 4 months and 1 year after the treatment. There were no grade 3 or higher toxicities.

## 4. Discussion

For a long time, the focus was on improving long-term oncological results; however, with oncologic results improving, quality of life and functional aspects have been gaining attention. Additional data from organ preservation strategies have contributed to a more conservative management strategy among the years. Habr Gama, a surgeon from San Paolo, was the first to pioneer this strategy and published good results in 2004 [15]. At this time, the results were widely discussed, and it was clearly not the route to take. French Greccar studies, Dutch studies, and British studies showed that this kind of strategy is feasible and safe for our patients [16,17,18]. The International Watch & Wait Database (IWWD) is a large registry with more than 800 patients in 15 countries. In this analysis of the outcome of patients treated conservatively, the 2-year cumulative incidence of local recurrences after a median FU of three years was around 30%, with most recurrences (88%) being diagnosed in the first two years [11]. In parallel, several reviews and meta-analyses on this topic have been published in recent years, reporting comparable overall survival and disease-specific survival rates to historical cohorts, allowing this conservative procedure to be considered oncologically safe in selected patients [19,20,21,22].

Unfortunately, despite these promising results, around one-fourth of patients experience relapse and will need surgery. When a Papillon boost is added to RT, higher rates of local control are achieved, reducing local recurrence by two-thirds [23,24]. In addition to older publications by Professor Papillon in France, who demonstrated the great ability of this technique to treat small polypoid tumors (LC = 91%) in the 1970s and 1980s [25,26], European centers have their unique experiences with Papillon as a single treatment of small polypoid tumors or in combination with CRT as part of a planned organ-preserving therapy [23,24,27,28,29]. For example, in a study conducted by Sun Myint, 86% of patients achieved a long-term (5-year) remission rate after treatment with CRT and with the addition of a Papillon boost [23]. Gerard also achieved a surprisingly high clinical complete response (96%) with high long-term organ preservation (89%, n = 112) [24]. More recently, a multicentric phase III study (OPERA) was initiated in 2015 with the aim of prospectively demonstrating the benefit of a Papillon boost. cT2/cT3 cN0/cN1 rectal carcinomas were randomized between conventional CRT +/− intracavitary contact RT boost. Patients in Arm A received a supplemental EBRT at a cumulative dose of 54 Gy. In Arm B, the boost was administered with Papillon (3 × 30 Gy). The patients were examined at intervals of 14, 20, and 24 weeks. If there was a complete clinical response, watch and wait (WW) was continued. Surgical intervention (TME or transanal resection) was performed in patients with an incomplete response or residual tumor. The results were recently published demonstrating a clear advantage of adding a Papillon boost. In fact, after a median FU of three years, the organ preservation rate was significantly higher in the Papillon arm: 81% versus 60% (*p* = 0.005). This benefit was even more striking for smaller tumors (97% versus 65%; *p* = 0.02) [30].

Our results are in line with the abovementioned outcomes and show a very high rate of local remission with sustained long-term organ preservation in well selected patients. All of our patients achieved a clinical complete response rate at the first assessment, which is of strong predictive value as it correlates with long-term organ preservation rate and survival outcomes [31]. In fact, only one of our patients needed major surgery (TME), and so far, half of our cohort have achieved long-term organ preservation. When relapse occurred (in 2 of our patients), it was in the rectum within the first 2 years, just like in the IWW database. As per large database sets, even if recurrence occurs, OS is not affected. Both of our patients were managed without compromising the chance of cure; one was offered salvage TME and the other underwent an endoscopic resection without sacrificing the organ. It was suggested that dose escalation with contact X-ray RT may negatively affect the outcomes of salvage surgery because of edema and fibrosis after RT. but recent data from the OPERA trial clearly do not confirm this hypothesis and show that TME can be safely performed after local dose escalation [32,33].

A prerequisite for a WW procedure is a well-conducted FU after CRT + Papillon boost have been completed. Since most recurrences occur in the first 2 years, the FU in these first two years is very intense for the patient. Several guidelines recommend a rectoscopy, DRE, and MRI every three months. The concern that patients pursuing a WW strategy are at higher risk for distant metastases does not seem to be confirmed. In fact, recurrence rate other than endoluminal in the IWWD was of only 3%

The use of total neoadjuvant treatment (TNT) for patients with unfavorable prognostic factors (cT3-T4, N2, EMVI+) was recently revolutionized by two randomized phase 3 trials [31,32]. Despite the benefit in terms of distant relapse, in both studies, the rate of pathologic complete responses was 25%, expanding the opportunity for OP. This becomes even more important in patients with lower tumors where an amputation is needed (22–38% in the abovementioned trials). Further encouraging data are provided from a recent phase 3 trial showing that OP is possible in about 50% of patients with a more advanced locoregional tumor when neoadjuvant systemic treatment is intensified [33]. So far, the WW approach is mainly reserved for patients presenting the early stages of cancer, but the results of these phase 3 trials provide us with good evidence that conservative management could be implemented to a wider population. Better results in terms of OP and downstaging can be achieved by combining intensified chemotherapy and a local dose escalation, notably with the Papillon.

The interest in avoiding surgery is not only based on the desire to avoid a definitive stoma but also to preserve bowel function, which is a major challenge especially in patients presenting with a low or ultra-low tumor. Bowel function maintenance is an important aspect, and patients treated with Papillon show a very high quality of life in terms of their bowel function [23,24,27,28,29,30]. Due to the particular physical properties described above, the acute and late toxicities associated with the use of Papillon are in fact very limited. Acutely, intermittent grade I proctitis, grade I rectal ulceration, and/or local grade I-II telangiectasia associated with mild, self-limiting hematochezia may occur in less than one-third of patients. Late toxicity is very rare; only a few cases of stenosis or fistula formation, exceptionally requiring colostomy, have been described in the literature. No mortality associated with Papillon toxicity has been reported. In our experience, toxicity was rare, and minor rectal bleeding reported in a minority of patients as a late side effect could be easily managed with local cauterization. In very low seated tumors, discomfort in the perianal region because of irritation of the anal mucosa can occur, but this is easily managed with minor analgesics and topical steroids.

Finally, in the multimodal treatment of rectal cancer, the economic burden of surgical treatment clearly outweighs that of conservative approaches. Despite the apparently expensive need for frequent FU care after WW, the costs for radical surgery, but also for colostomy and the treatment of complications, are still significantly higher [34]. In Switzerland, the cost of a Papillon treatment with three fractions is approximately CHF 3000, which is up to 10–20 times lower than the cost directly associated with an abdominal–perineal resection of the rectum.

## 5. Conclusions

The current paradigm for rectal cancer treatment is evolving rapidly. It is very important to adapt the treatment method to each individual patient, with quality of life and functional aspects playing a very important role.

With current CRT, about a quarter of patients achieve complete clinical remission. These patients are good candidates for a WW procedure without jeopardizing their survival. Unfortunately, the recurrence rate is still relatively high at 30%. Long-term local control and recurrence rates can be significantly improved with a Papillon boost. Selected local recurrences can also be treated using this method in an attempt to preserve the organ and avoid the side effects and expenses of surgery. Our experience with Papillon shows, in our Swiss cohort of patients, a high rate of complete local response, allowing these patients to achieve long-term organ preservation. Our results are in line with the recently proffered 3-year results of the OPERA randomized trial. This unique treatment modality may help future patients with rectal cancer to benefit from low toxicity RT dose escalation, achieve complete local response, and avoid surgery. Detailed patient information, interdisciplinary cooperation, precise staging, and careful FU are prerequisites for a successful outcome.

## Figures and Tables

**Table 1:** Baseline patient, disease and treatment characteristics.

	No.
**Median age (range), years**	70 (49–91)
**Sex**	
Male	19
Female	5
**ASA score**	
I	15
II	8
III	1
IV	0
**AJCC 8 stage**	
I	11
II	7
III	5
IV	1
**T classification**	
T1	4
T2	9
T3	10
T4	1
**N classification**	
N0	19
N1	4
N2	1
**M classification**	
M0	23
M1	1
**Localization**	
Middle (6–10 cm)	4
Low (up to 5 cm)	16
Ultra-low (touching dentate line)	4
**EBRT**	
Alone	4
With CCT	20
**CRT dose, Gy**	
90	12
<90	12

Abbreviations: ASA: American Society of Anesthesiologists (ASA) physical status classification system, AJCC: American Joint Committee on Cancer, EBRT: external beam radiotherapy, CCT: concomitant chemotherapy.

**Table 2:** Summary of CXB-related adverse events: acute (<3 months).

Adverse Event	Grade 1 Number (%)	Grade 2 Number (%)	Grade ≥ 3 Number (%)
Gastrointestinal			
Erratic bowels	9 (37%)	0	0
Bloating	2 (8%)	0	0
Mucous discharge	9 (37%)	0	0
Perianal/rectal Pain	4 (17%)	4 (17%)	0
Rectal bleeding	5 (22%)	0	0

CXB: contact X-ray radiotherapy.

**Table 3:** Summary of CXB-related adverse events: late (>3 months).

Adverse Event	Grade 1 Number (%)	Grade 2 Number (%)	Grade ≥ 3 Number (%)
Gastrointestinal			
Erratic bowels	1 (4%)	0	0
Mucous discharge	2 (8%)	0	0
Perianal/rectal Pain	0	0	0
Rectal bleeding	1 (4%)	3 (13%)	0

CXB: contact X-ray radiotherapy.

## Data Availability

The original contributions presented in the study are included in the article. Further inquiries can be directed to the corresponding author.
